# Towards a Holistic Understanding of Musician’s Focal Dystonia: Educational Factors and Mistake Rumination Contribute to the Risk of Developing the Disorder

**DOI:** 10.3389/fpsyg.2022.882966

**Published:** 2022-05-09

**Authors:** Anna Détári, Hauke Egermann

**Affiliations:** ^1^York Music Psychology Group, Department of Music, University of York, York, United Kingdom; ^2^Institut für Musik und Musikwissenschaft, Technische Universität Dortmund, Dortmund, Germany

**Keywords:** musician’s focal dystonia, psychosocial risk factors, mistake rumination, holistic model, music education

## Abstract

Musicians’ Focal Dystonia (MFD) is a task-specific neurological movement disorder, affecting 1–2% of highly skilled musicians. The condition can impair motor function by creating involuntary movements, predominantly in the upper extremities or the embouchure. The pathophysiology of the disorder is not fully understood, and complete recovery is extremely rare. While most of the literature views the condition through a neurological lens, a handful of recent studies point out certain psychological traits and the presence of adverse playing-related experiences and preceding trauma as possible contributors to the onset. The nature and the frequency of these factors, however, are under-researched. The present quasi-experimental study aimed to compare musicians with and without MFD in terms of the frequency of various adverse psychosocial and psychological factors to explore their contribution to the onset of the condition. Professional musicians with MFD (*n* = 107) and without MFD (*n* = 68) were recruited from online platforms, musicians’ unions, and organisations to fill out a survey. The survey was based on two previously conducted interview studies and included the Student-Instructor Relationship Scale (SIRS), the Mistake Rumination Scale (MRS), the Trauma History Screen, and self-constructed questions about the received music education, early success, and personal experiences. To identify potential risk factors, independent samples *t*-tests were conducted and found that there are significant differences in musicians with and without MFD in terms of mistake rumination, early success, and the received music education. A logistic regression showed that six factors contributed to the construct to various extents; we observed a significant model [χ^2^_(80)_ = 22.681, *p* < 0.001], which predicted 71.2% of the cases correctly. This exploratory study shows that psychological and psychosocial factors might play a role in the development of MFD. Understanding these in more detail could inform preventative strategies and complement the current therapeutic approaches to support this vulnerable population better.

## Introduction

In the past decades, there is a growing awareness of the occupational problems and disorders of performing musicians (Schuele and Ledermann, 2004; Kok et al., 2016). Among these, Musician’s Focal Dystonia (MFD), a task-specific neurological movement disorder, seems to be the most elusive in terms of origins and triggering factors. The condition impairs the fine motor control of body parts that contribute to the sound production, most frequently the upper extremities and the embouchure (Jankovic and Ashoori, 2008; Altenmüller and Jabusch, 2010; Termsarasab and Frucht, 2016), but sometimes even affects the lower extremities (Lee and Altenmüller, 2014). The symptoms manifest themselves in the form of cramps, tremors, and tension in specific muscle groups, and result in involuntary movement patterns. MFD appears to be highly task-specific with only a small percentage of sufferers developing non-task-specific symptoms (Hofmann et al., 2015). It is estimated that it affects about 1–2% of highly accomplished musicians (Altenmüller and Jabusch, 2010), however, this number might be even higher because it is challenging to diagnose the disorder, moreover, some musicians might not seek medical help and their problem remains undiscovered (Rosset-Llobet et al., 2009; Sussman, 2015).

While our understanding evolved greatly of the genetic predisposition of the sufferers (Schmidt et al., 2009) and the maladaptive neurological changes that take place (Elbert et al., 1998; Byl et al., 2000; Candia et al., 2003, 2005; Haslinger et al., 2010), the aetiology it is still unclear (Altenmüller and Jabusch, 2009) which makes the treatment and the development of preventative strategies challenging.

To clarify the origins and the multifactorial network of triggering factors, researchers broadened the field of inquiry by exploring the individual characteristics of the sufferers and found that certain psychological traits, cognitive patterns, and practice behaviours might be contributing factors in the onset (Altenmüller et al., 2014; Sadnicka et al., 2018). A string of studies showed that musicians with MFD have higher levels of anxiety, perfectionism, and social and other types of phobias than healthy musicians and musicians with chronic pain (Jabusch and Altenmüller, 2004; Jabusch et al., 2004; Enders et al., 2011), and it has been concluded that these are pre-existing characteristics rather than psychoreactive traits responding to the onset (Altenmüller and Jabusch, 2009; Enders et al., 2011).

Anxiety, perfectionism, and phobias are closely associated with maladaptive cognitive strategies, such as overfocusing and reinvestment, which were also suggested as triggering factors (Altenmüller et al., 2014), and are known to interfere with the execution of motor movements in various settings (Wulf, 2013), including music performance (Duke et al., 2011; Mornell and Wulf, 2019). It is plausible that these characteristics informed the practice behaviours of the musicians, prompting over-involvement in the task, to the level of motor fatigue and overuse injuries, which can be initial indicators of the onset as well (Altenmüller et al., 2014).

All of these possible psychological, cognitive, and behavioural contributing factors are usually discussed in the literature as highly individual traits and behaviours; however, they do not develop in a vacuum. The social and cultural context, in which the individual is embedded plays an important role in the development and cultivation of the personality and behaviours (Wulf and Lewthwaite, 2016), therefore, these external (sociocultural and psychosocial) influences should not be overlooked.

As an example, there is a rich literature on how social expectations can increase maladaptive perfectionism (Damian et al., 2013), especially in the context of close and influential relationships, such as family systems (Rasmussen and Troilo, 2016). The phenomenon has been examined in sports settings, due to the close relationship between athletes and coaches, and the findings show that coach pressure is an even stronger indicator of long-term perfectionism than parental pressure (Madigan et al., 2019). In instrumental tuition, the student is similarly reliant on their instrumental teacher as an athlete on their coach: the tuition is delivered individually, most often following a master-apprentice model (Haddon, 2009), so much so that instrumental teachers have been called musical parents (Creech and Hallam, 2003). Therefore, it is possible that the frequently reported perfectionism of MFD sufferers is, at least partially, induced or reinforced in the educational setting. Mental problems, such as heightened levels of anxiety also develop in a social and cultural context and were argued to be “functional responses to adversity” (Syme and Hagen, 2020, p. 104) rather than just maladaptive innate traits or inherited conditions. Reinvestment and over-focusing can also be prompted by external factors; these cognitive strategies intensify when there is a perceived danger of being negatively evaluated (Masters et al., 1993). In other words, these characteristics in musicians with MFD might be aggravated by the demands of the professional environment.

The frequently mentioned over-involvement in practice which often results in overuse and motor fatigue in this population (Altenmüller et al., 2014), can be prompted by the aforementioned psychological traits and cognitive patterns, but again, the professional environment possibly also plays a role. It seems logical that the instrument-related behaviours are learned in the instrument-related context, and the performance and practice behaviours are modelled from the specific environment, presumably from the teachers and peers.

The idea of external factors influencing the onset of the condition is further reinforced by the fact that the onset often follows a traumatic experience (Tubiana, 2003). To the best of our knowledge, there is no in-depth investigation into the nature of these events and how they influence the individual, but there is some evidence that many musicians encounter a triggering incident that elevates stress levels before the first symptoms appear (Schmidt et al., 2013).

Yet, the majority of the literature considering psychological and behavioural factors in the onset largely neglects how these characteristics evolved in musicians. These features are examined in isolation, with little regard for the highly specialised environment in which they developed. This leads not only to a limited understanding of the condition but also, perhaps unknowingly, results in a narrative that holds the sufferers at least partially responsible for their condition, by highlighting their maladaptive psychological and behavioural traits. Liley (2019), when examining the narrative around injured pianists, concluded that the negative portrayal of the sufferers is counterproductive in terms of supporting the population and steers the conversation away from important contributing factors, such as the quality of the received training. Therefore, it is important to understand what shaped these musicians’ behaviours, cognitions, and even emotions in connection to their instruments, music-making, and careers.

To explore this under-researched area, we initially approached the topic with an open and qualitative methodology (Détári et al., 2022) to gather data directly from the source, namely, the personal experiences of MFD sufferers, which was followed by a semi-structured interview study with practitioners who treat the condition frequently (Détári and Egermann, in press). These studies reinforced the hypothesis that some of the frequently cited characteristics of this population might develop under the influence of external factors.

Given that the symptoms of MFD evolve and are experienced in a professional setting, most often affecting only the movements associated with performance (Hofmann et al., 2015), it was quite unsurprising that the findings were closely associated with the educational context and work environment. The data from these studies show that the musicians with MFD had unfavourable experiences with their music tuition: they were exposed to socially prescribed perfectionism and authoritative teaching styles in a strict, sometimes even abusive social environment, which expressed little or no regard for performance-related pain or injury. This often prompted anxiety, perfectionism, and mistake rumination in the participants, which led to unhealthy practice behaviours.

The aim of the present study is to examine the topic further by testing the hypotheses generated through the two qualitative interview studies (Détári and Egermann, in press; Détári et al., 2022) in a large-scale quasi-experimental study. We want to identify the most frequent risk factors within the MFD population by comparing them with a control group of healthy musicians without MFD. The goal was to understand how the educational settings, the professional environment, and cognitive strategies might contribute to the development of the condition. The findings can have important implications for music education and for developing more efficient preventative strategies.

## Materials and Methods

### Design, Setting, and Participants

The questionnaire study is the final stage of a larger mixed-method study. The previously conducted interview studies with MDF sufferers (Détári et al., 2022) and practitioners (Détári and Egermann, in press), which retrospectively explored the personal experiences of MFD sufferers prior to the onset, served as a basis for the present survey study. The topics were selected from this rich qualitative material and aimed to cover themes that were strongly supported by both interview studies and were unexplored by previous literature. We also attempted to link this content to previously established concepts and use validated scales when available.

The questionnaire was administered online, and apart from identifying as a musician with or without MFD, we set no exclusion criteria. Participants were recruited from online support groups and social media platforms, and various organisations and practitioners were asked to share the information and the links in their networks. A total of 240 musicians (125 with MFD and 115 healthy) answered the survey. After omitting unfinished and partial responses, 175 cases were considered for further analysis. To allow for a more precise comparison, a pairwise matching between participants with and without MFD was conducted based on gender, age, and instrument group, which resulted in a subset of 88 musicians. Information about the mean age, gender ratio and instruments played in the sample and the subset is presented in Tables 1, 2.

**TABLE 1:** Age and gender characteristics of the samples.

Group	Age	Gender distribution			
	*M* (SD)	Female	Male	Non-binary	Prefer not to say
Full sample (*N* = 175)	42.5 (14.3)	79 45.1%	93 53.1%	1 0.5%	2 1.1%
Full MFD sample (*N* = 105)	44.4 (14.2)	40 38%	63 60%	0 -	2 1.9%
Full healthy sample (*N* = 70)	39.5 (13.9)	39 55.7%	30 42.8%	1 1.4%	0 -
Selected subset (*N* = 88)	42.3 (13.5)	46 52.2%	42 47.7%	0 -	0 -
Selected MFD cases (*N* = 44)	42.5 (13.6)	23 52.2%	21 47.7%	0 -	0 -
Selected healthy cases (*N* = 44)	42.1 (13.6)	23 52.2%	21 47.7%	0 -	0 -

*The standard deviation is presented after the means in parentheses. The percentages are calculated in relation to the group size which is presented in the first column. MFD = Musician’s Focal Dystonia.*

**TABLE 2:** Instrument distribution of the samples.

Group	Instrument distribution					
	Wind	Brass	Strings	Percussion	Piano	Plucked
Full sample (*N* = 175)	42 24%	44 25.1%	20 11.4%	8 4.5%	33 18.8%	28 16%
Full MFD sample (*N* = 105)	22 20.9% (8–7.6% with ED)	30 28.5% (25–23.8% with ED)	8 7.6%	6 5.7%	17 16.1%	22 20.9%
Full healthy sample (*N* = 70)	20 28.5%	14 20%	12 17.1%	2 2.8%	16 22.8%	6 8.5%
Selected subset (*N* = 88)	26 29.5%	18 20.4%	18 20.4%	4 4.5%	12 13.6%	10 11.3%
Selected MFD cases (*N* = 44)	13 29.5% (3–6.8% with ED)	9 20.4% (9–20.4% with ED)	9 20.4%	2 20.4%	6 13.6%	5 11.3%
Selected healthy cases (*N* = 44)	13 29.5%	9 20.4%	9 20.4%	2 20.4%	6 13.6%	5 11.3%

*MFD, Musician’s Focal Dystonia; ED, Embouchure Dystonia.*

### Study Variables

We collected sociodemographic information (age, gender, instrument played, level of education, and profession), and questions were administered about the participants’ experience with the received music education, performance-related mistake rumination, lifetime prevalence of trauma, and other stress-inducing experiences. Additionally, we inquired about the location of the symptoms of MFD and the received diagnosis in the MFD group.

### Instruments

The first main topic of the investigation was the experience with the received music education. In the absence of a measurement tool specifically developed for this construct, we choose to use the Student-Instructor Relationship Inventory (SIRS) (Creasy et al., 2009; Creasey et al., 2009) and asked the participants to report about one instrumental teacher with whom they worked with and found most influential. The SIRS measures the relationship between the student and teacher on an anxiety-connectedness axis and has been reported to have good psychometric properties (α = 0.87 in the anxiety factor and α = 0.92 in the connectedness factor) (Creasy et al., 2009). Additionally, it has the benefit of targeting college-level students–the age group where most of the professional teaching is happening–making this measurement tool even more appropriate. Since there were many topics in the interview studies relating to the profession-specific aspects of instrumental teaching, we added self-constructed questions to the survey. These were aimed at the content of the received teaching, with topics like received information about healthy playing technique and performance-related injuries, authoritative teaching style, socially prescribed perfectionism, and technique-focused teaching. In addition, following the qualitative data, we also included questions about the participants’ early accomplishments and the difficulty of the played material in relation to their peers. The 24 self-constructed items are presented in Appendix A (Supplementary Material).

We also inquired about performance-related mistake rumination using the Mistake Rumination Scale (MRS) (Flett et al., 2020), which was strongly supported by the qualitative data. This scale was tested on five samples (Flett et al., 2020) and was found to be valid to measure the construct (α > 0.81 and all items loading 0.50 or greater in all samples).

Data was collected about the experienced trauma over the lifetime with the Trauma History Screen (Carlson et al., 2011), and additional self-constructed questions were added about significant changes in the participants’ lives. These included events listed by the participants in our interview study (Détári et al., 2022), such as changing instruments, teachers or workplaces, and other personal events, such as becoming a parent or getting divorced. These potential risk factors and all scales employed are presented in Table 3.

**TABLE 3:** Topics and scales.

Topic	Used scales
Experiences with music education	Student-Instructor Relationship Inventory (SIRS) (Creasy et al., 2009)
	Self-constructed questions (about teaching style, body mechanics, technique-focused teaching, and early success)
Mistake rumination	Mistake Rumination Scale (MRS) (Flett et al., 2020)
Trauma and change	Trauma History Screen (Carlson et al., 2011)
	Self-constructed questions

### Procedure

After the participants received a link to the questionnaire, they were presented with a downloadable information sheet and were asked to give consent to the research team to use their anonymised data. Without consent, they could not proceed to the survey questions. The survey was open for 2 months.

### Data Analysis

For data analysis, SPSS and R software were used. First, responses with more than three data points missing were omitted manually. In the following analyses, the remaining missing values were replaced by means. The open questions regarding the instruments played were dummy coded twice, firstly, all different instruments were assigned a different number, and secondly, the instruments were grouped into six categories (woodwind, brass, string, piano, percussion, and plucked instruments) with one assigned number for each category. The location of the MFD symptoms in the group with MFD was also coded into three categories (upper extremities, embouchure, and other). Following this, descriptive tests were run to explore the sociodemographic variables.

In the second stage of the analysis, the instruments were inspected: Exploratory Factor Analyses (EFAs) and Confirmatory Factor Analyses (CFAs) were run on the scales to evaluate them and to test their internal consistency.

To map the differences in risk factors between the groups, we conducted *t*-tests on each variable and tested the relationship between them with a Pearson r correlation. In order to create a comprehensive model of the variables, a logistic regression was used using the presence of MFD as a dependent variable. A detailed description of the tests and the procedure are presented in the result section.

### Ethical Considerations

Musicians who suffer from dystonia are vulnerable to psychological distress when asked to recall details about the condition which endangers both their livelihood and artistic outlet. The information sheet and accompanying documents were phrased with this in mind, avoiding negative language, and providing resources of support at the conclusion of the survey. Ethical approval was obtained from the Arts and Humanities Ethics Committee at the University of York, United Kingdom.

## Results

### Descriptive Statistics of Participant Sample Characteristics

The average age of musicians with MFD (*N* = 105) was 44.46 years (SD = 14.29) ranging from 21- to 83-year-old, and the average age of the onset was 35.57 years (SD = 16.743). They started playing the instrument at 13.09 years of age (SD = 7.14), although this number is skewed by some amateur players, who started playing between the ages of 35–50. After removing these outliers, the average age of starting instrumental education was 12.20 years. The participants suffered from the onset of MFD after 21.13 years of playing on average (SD = 13.491). 64.7% of the musicians considered themselves professionals with a further 15.2% responding “maybe,” and 20% of the sample was amateurs. Responses to the open question added at this point suggested that many professional musicians reported “maybe” because they were unable to pursue their profession full time due to their symptoms at the time of their responses. 63.8% of the musicians were diagnosed by a neurologist, further 9.5% by another practitioner, 4.7% by fellow sufferer or musician, and 21.9% were self-diagnosed. There were 31 participants (29.5%) with embouchure problems in the sample with the rest reporting problems with their upper extremities, most often their fingers.

### Factor Analytic Scale Construction

#### Self-Constructed Music Education Scale

20 items were examined for factorability. Half of the items were worded to express the opposite of the targeted construct to avoid participant bias; the scores for these items were reversed for the analysis. The Kaiser-Meyer-Olkin measure of sampling adequacy was 0.788, above the commonly recommended value of 0.6, and Bartlett’s test of sphericity was significant [χ^2^_(190)_ = 1199.250, *p* < 0.001]. All items correlated at least with one other (>0.3), showing that each item had some level of common variance with the other items, which suggested acceptable factorability. Table 4 presents the results of an Exploratory Factor Analysis with Promax rotation, using parallel analysis (Vivek et al., 2017) and loading values below 0.40 were suppressed (Hair, 2009). For this procedure, we used the full sample (*N* = 175); the higher number of responses provided more data to establish the factors.

**TABLE 4:** Factor loadings–EFA pattern matrix “music education”.

	Low health support and encouragement	Technique focused teaching	High teacher demands and authoritative style
Tech		0.53	
No posture	0.79		
Own technique (Rev.)		0.79	
Expression (Rev.)	0.46		
Avoid injuries (Rev.)	0.76		
Unsatisfied			
Too much asked			0.59
First try			0.68
No health support	0.55		
Give time (Rev.)	0.66		
Unsure expectations			
Prescription			0.74
Clear instructions (Rev.)	0.62		
Encouragement (Rev.)	0.53		
Concern over discomfort (Rev.)	0.74		
Technical difficulty		0.47	
Solving issues (Rev.)	0.80		
Changed technique		0.81	
Own approach (Rev.)		0.41	
Self-efficacy (Rev.)			

Three underlying factors were identified, Low health support and encouragement, Technique focused teaching, and High teacher demands and authoritative style, explaining 27.1, 12.9, and 7.7% of the variance respectively. Two items were omitted because they did not meet the criteria of minimum loading of 0.40, and one because it cross-loaded on two factors. A confirmatory factor analysis was subsequently conducted on the remaining items and reinforced that the factor model was a good fit [χ^2^_(113)_ = 345.861, *p* < 0.001], and the three factors showed acceptable internal consistency (α = 0.783, α = 675, α = 757, respectively). This CFA model was used to calculate the factor scores for each factor and each participant, and these scores were used in the subsequent analysis. The items in the three factors are presented in Appendix B (Supplementary Material).

#### Early Success Scale

A second factor analysis was conducted on the items relating to early success on the instrument. The items were moderately correlating with each other (0.453–0.666) showing common variance, but uniqueness. Kaiser-Meyer-Olkin measure of sampling adequacy (0.767) and the Bartlett’s test of sphericity [χ^2^_(6)_ = 248.514, *p* < 0.001] showed that the items met the requirements for factor analysis. The EFA was run on the full sample (*N* = 175), with the use of parallel analysis and Promax rotation, and with the sample size and degrees of freedom in mind, the loading values below 0.40 were suppressed. The analysis and the following CFA showed one underlying factor clearly accounting for 65.4% of the variance with good internal consistency (α = 0.818) and was dubbed as “Early success.” The loading table and the correlation table are presented in Tables 5, 6.

**TABLE 5:** Loading table–EFA pattern matrix, “early success”.

	Component 1.
Easy start	0.770
Early success	0.765
Quick improvement	0.866
Ahead of class	0.831

**TABLE 6:** Pearson r correlation matrix “early success”.

	Easy start	Early success	Quick improvement	Ahead of class
Easy start	1.000			
Early success	0.45	1.000		
Quick improvement	0.60	0.51	1.000	
Ahead of class	0.46	0.54	0.67	1.000

Two further CFAs were run on the selected items of the SIRS and the modified Mistake Rumination Scale. The CFAs reinforced the two underlying factors in the SIRS: anxiety around the tutor (α = 0.913) and connectedness to the tutor (α = 0.922) [χ^2^_(103)_ = 337.490, *p* < 0.001], and one underlying factor in the Mistake Rumination Scale (α = 0.897) [χ^2^_(14)_ = 60.026, *p* < 0.001]. In both scales, means were used as scores for each participant and each factor in the subsequent analysis.

### Comparing Risk Factors of Matched Samples of Musicians With and Without Musicians’ Focal Dystonia

Independent samples t-tests were conducted to identify mean differences between both sub-samples (Healthy vs. MFD, *n* = 88) in all potential risk factor variables (Table 7). The analysis showed that musicians with dystonia suffered from more performance-related mistake rumination and had significantly more success in their early careers. In addition to this, they had significantly lower health support and encouragement (Factor 1.) and more demands were placed on them with an authoritative teaching style (Factor 3.).

**TABLE 7:** Comparison between musicians with and without musicians’ focal dystonia (MFD).

Risk factor variable	Healthy *M* (SD)	MFD *M* (SD)	*t*	df	*p*	Cohen’s *d*
SIRS Anxiety around tutor	2.40 (0.93)	2.78 (1.06)	1.78	86	0.078	0.28
SIRS Connectedness to tutor	3.74 (0.88)	3.49 (0.87)	1.35	86	0.180	0.38
Mistake rumination	2.17 (0.72)	2.75 (0.85)	3.45	86	<0.001***	0.73
Low health support and encouragement (Factor scores for factor 1)	–0.27 (0.90)	0.10 (0.77)	2.12	86	<0.005**	0.45
Autonomy in instrumental technique (Factor scores for factor 2)	–0.15 (0.96)	0.12 (0.99)	1.33	86	0.184	0.28
Demands and authoritative teaching (Factor scores for factor 3)	–0.11 (0.88)	0.24 (0.75)	2.03	86	<0.005**	0.43
Early success	3.30 (1.00)	3.92 (1.02)	2.83	86	0.006**	0.60
Trauma frequency	1.81 (2.04)	1.95 (1.79)	0.33	86	0.740	0.07
Significant life event frequency	2.04 (1.65)	2.52 (1.54)	1.39	86	0.166	0.29

Standard deviations are presented in parentheses. SIRS, Student-Instructor Relationship Scale; MFD, Musician’s Focal Dystonia; **p < 0.01, ***p < 0.001.

### Correlations Between Risk Factor Variables

To test the shared variance in the risk factor variables a correlation table was created which shows a strong correlation between the self-constructed scale’s factors (Health and encouragement, Autonomy in instrumental technique, Demands and authoritative teaching) and the SIRS scale’s “anxiety around tutor” factor (Table 8). Moreover, there is a strong negative correlation between connectedness to the tutor (SIRS scale) and the first factor (Health and encouragement) of the self-constructed scale, and a positive correlation between anxiety around the tutor (SIRS scale) and the mistake rumination scale.

**TABLE 8:** Pearson r correlation matrix for risk factor variables.

Variable	SIRS connect	SIRS anxiety	Mist. ruminat.	Low health supp.	Autonomy	Demands	Early success	Trauma
SIRS connect	–							
SIRS anxiety	–0.54*							
Mist. ruminat.	–0.14	0.48**						
Low health supp.	–0.68**	0.44**	0.17					
Autonomy	–0.16	0.51**	0.26*	0.12				
Demands	–0.09	0.52**	0.37**	0.18	0.22*			
Early success	–0.14	0.22	0.13	0.12	0.01	0.06		
Trauma	0.15	0.16	0.21*	–0.12	0.07	0.15	0.10	
Change	–0.05	0.24*	0.27*	–0.002	0.15	0.26*	0.29*	0.28**

SIRS = Student-Instructor Relationship Scale; probability values are calculated, and r correlations marked with * where values lower than 0.005 and with ** where values lower than .001.

### Logistic Regression

Considering the rather frequent moderate to high correlations between risk factor variables, we subsequently run a logistic regression on the matched subsample of MFD sufferers and healthy musicians (*n* = 88), in order to identify the most relevant risk factors for developing MFD. As an outcome variable, we coded being affected by MFD with 1 and not being affected with MFD 0. All nine predictor variables were added to the model: anxiety around the tutor (SIRS scale, factor 1), connectedness to the tutor (SIRS scale, factor 2), performance-related mistake rumination, Health and encouragement (self-constructed scale, factor 1), Autonomy in instrumental technique (self-constructed scale, factor 2), Demands and authoritative teaching (self-constructed scale, factor 3), early success, trauma frequency, and significant life event frequency. We also included the age and gender variables in the model to control for their possible effects. Following this, in order to simplify the model and to avoid over fitting, risk factor variables with Wald statistics lower than 1 (SIRS connectedness to the tutor, trauma frequency, change frequency, age, and gender) were excluded from the analysis in a backwards fitting approach.

The results indicate that there are two highly influential, significant risk factor variables contributing to the construct: Mistake Rumination and Early success, two variables showing non-significant trends and two non-significant variables where the estimates show the predicted direction. With the inclusion of these risk factor variables, we observed a significant model ([χ^2^_(80)_ = 22.681, *p* < 0.001], Nagelkerke *R*^2^ = 0.306), which predicted 71.2% of the cases correctly. The coefficient table is presented in Table 9.

**TABLE 9:** Musicians’ focal dystonia (MFD) risk factor coefficients from logistic regression.

	Confidence interval				Wald test		
	Coefficient estimate	Odds ratio	Lower bound	Upper bound	Wald statistics	df	*p*
(Intercept)	–2.78	0.06	–5.29	–0.28	4.74	1	0.029
Mist. ruminat.	0.94	2.56	0.24	1.64	6.95	1	0.008
Early success	0.66	1.94	0.16	1.16	6.82	1	0.009
Low health supp. (F.1.)	0.63	1.89	–0.036	1.31	3.44	1	0.064
SIRS anxiety	–0.72	0.48	–1.53	0.09	2.98	1	0.084
Autonomy (F.2.)	0.37	1.45	–0.21	0.96	1.55	1	0.212
Demands (F.3.)	0.43	1.55	–0.25	1.12	1.54	1	0.213

*Group with MFD coded as class 1. SIRS, Student-Instructor Relationship Scale.*

## Discussion

The present study approached the question of the aetiology of MFD from a new perspective, placing it in a broader context. We concluded that there is evidence that apart from the frequently cited genetic predisposition (Schmidt et al., 2009), personality traits, maladaptive cognitive strategies, and overuse (Altenmüller and Jabusch, 2009; Enders et al., 2011; Altenmüller et al., 2014; Sadnicka et al., 2018), external social factors might also play a part. We found significant differences between musicians with MFD and healthy musicians in terms of the education they participated in; musicians with MFD were more likely to receive less information about healthy playing technique, higher demands were placed on them with an authoritative teaching style, were less encouraged, and excelled at their instrumental studies, playing more demanding materials than their peers. We also added performance-related mistake rumination to the already suggested maladaptive cognitive strategies and have shown that it is a potential risk factor. Two of these factors were highly significant contributors to the logistic regression model, mistake rumination and early success; other educational factors showed significant differences between the groups in the *t*-tests and were not-significant trends, showing the predicted direction in the regression model.

Given that MFD is a highly task-specific condition (Hofmann et al., 2015), it seems difficult to fully understand it without considering the context in which the affected skill was learned, practised, and performed. The process of movement acquisition can provide further clues for understanding the condition’s aetiology, moreover, it might help us to understand the task-specific nature of the disorder; a characteristic that the current models cannot fully explain (Sadnicka et al., 2018).

### Playing Mechanisms

Much has been written about how years of excessive repetition of the motor skill is a prerequisite for being affected by MFD: the onset usually happens to trained professionals after many years of practice and performance, most often in their mid-30s (Altenmüller and Jabusch, 2010; Sussman, 2015). However, what kind of movement patterns are being repeated, i.e., the quality of the playing mechanisms might be equally important as the quantity of the practice hours. Many musicians practise excessively without being affected by MFD; one explanation for this is the presence of genetic predisposition (Schmidt et al., 2009), however, it is possible that repeating less efficient movement patterns might also play a role. The quality, i.e., the way how the playing task is executed is largely determined by how it was learned in the first place. Our investigations into the educational context show that there are some distinct characteristics of the received tuition of musicians who later were affected by MFD.

Training professional musicians requires a special setting that largely differs from traditional classroom education. Through the frequently delivered individual sessions, the instrumental teacher gains a profound influence over a limited number of students (Gaunt, 2011; Patston, 2014). This master-apprentice model of teaching happens in relative isolation (Haddon, 2009), is mostly unsupervised, and has no in-built mechanisms for quality control (Kemp, 1996). The content of the teaching is often the replication of the teacher’s own education or personal experience (Mills and Smith, 2003; Visentin et al., 2008), and the centre of the attention is the musical output with much less attention to the physical movements or the posture the musicians use to create it (Chan and Ackermann, 2014). Moreover, music educators are often not well-informed about anatomically correct and instrument-specific body mechanics (Visentin et al., 2008), so students’ unhealthy postures and inefficient techniques might be undetected and unchallenged, especially if the individual is still able to produce the expected output. As a prominent example, Glenn Gould played flawless concerts with a clearly distorted and unhealthy posture, and while he had not been diagnosed with MFD in his lifetime, the movement patterns observed in video recordings led experts to believe that he was affected by the condition (Wilson, 2000).

What is clear, however, is that inefficient playing mechanics can overload the joints and the musculature, and lead to physical problems (Chan and Ackermann, 2014). There are strong established links between playing-related musculoskeletal disorders, overuse, and unhealthy postures in musicians (Steinmetz et al., 2010), and overuse has been proposed as a triggering factor for MFD as well (Baur et al., 2011; Altenmüller et al., 2014). This suggests that the biomechanical quality of the playing technique might play a part in developing the condition.

Results from the self-constructed scale’s first factor, “low health support and encouragement” shows the tendency that teachers of the musicians who later developed MFD cared less about educating their students about healthy playing technique and correct posture and were less concerned about performance-related pain or injury. We know that pain or discomfort experience can modify established movement patterns (Sterling et al., 2001) to avoid pain- or discomfort-triggering movements, moreover, the intensified somatosensory input may “lead to a degradation of sensorimotor representations at several levels of the sensorimotor circuits” (Altenmüller and Jabusch, 2009, p. 146). Fatigue and pain can also lower the motor system’s capacity to meet the demands of the performance. As Sadnicka et al. (2018) noted, these alterations of muscle recruitment, which can be inappropriate and inefficient, can have neurological consequences: if the neural representation of the skill cannot accommodate the new movement pattern, the performance will be impaired. In other words, unhealthy playing mechanisms, especially the ones which lead to pain or injury, can lead to developing compensatory movement patterns, and simultaneously, neurological changes. Unfortunately, many musicians keep playing with pain and injury, furthering this deterioration, due to the “no pain, no gain” culture among professional musicians and the stigma around performance-related pain which discourages musicians to seek help for their discomfort or pain (Ackermann, 2017). The primary music teacher’s role in compensating for this negative message is extremely important because they can have a profound effect on their students’ health and instrument-related behaviours, and at a younger age, students are more malleable to change maladaptive habits (Cruder et al., 2020).

### Early Success

Apart from the decreased motor capacity following pain, there are other situations in which the young musician is challenged to play more difficult materials they are capable of: during the learning process. While the instrumental technique is still developing, choosing an adequate repertoire, which requires enough challenge for development but does not overwhelm the student, is extremely important (Patston, 2014). If the developmental trajectory of the student is not kept in sight, and the student is pushed over the boundaries of normal motor development, a similar discrepancy between skill and demand presents itself, which can pose a risk for MFD (Sadnicka et al., 2018).

In our dataset, musicians with dystonia reported significantly more early success (i.e., playing more demanding materials than their peers and facing challenging performance opportunities). Assigning difficult materials to students might be due to misinterpreted levels of capability from the teachers’ part, but it is also possible that these young students showed great aptitude. This idea is further reinforced by the fact that musicians affected by the condition are often soloists (Lim and Altenmüller, 2003; Sussman, 2015) or playing in other esteemed positions, which suggests outstanding talent.

Also, the rapid advancement in playing the instrument might also be linked to the age these musicians began their instrumental studies: the average starting age for our participants with dystonia was 12.20 years (after removing the amateur outliers who started over 35). Children’s learning strategies undergo certain changes as their metacognition and verbal working memory develop. Very young children learn based on mimicking and repeating: the process is mostly unconscious and is not accompanied by cognitive verbal processes (Masters et al., 2013). This is called implicit learning or external focus of attention in the literature, and it produces robust, and rapidly automatised movement patterns, underpinned by neural efficiency (Zhu et al., 2014). Examples of these might be walking, manipulating objects, or riding a bicycle. As the child develops a more and more substantial capacity for metacognition, more cognitive skills are used in the process: conscious, analytical planning and testing out certain movement patterns. This is an explicit way of approaching the task at hand, with more conscious control involved in the process. This change in the way of learning is dependent on the task and there are individual differences, however, the ability rarely develops fully before the age of 11 (Gathercole et al., 2004; Masters et al., 2013). The fact that the average starting age in the sample was 12.20 years, and the literature reported similar findings across several samples of musicians with MFD (Schmidt et al., 2013; Altenmüller et al., 2014) suggests that musicians who later developed MFD employed a more explicit strategy, and more internal focus when acquiring the skill. While the literature clearly shows the superiority of implicit learning or external focus (Wulf, 2013) providing evidence that learning with an external focus enhances efficiency (Zachry et al., 2005), effectiveness (Wulf and Lewthwaite, 2010), technical precision, and musical expressivity in music performance (Mornell and Wulf, 2019), the findings regarding the attention of focus in the early stages of learning have been so far inconclusive (Stambaugh, 2017). It has been hypothesised that children with high motor ability benefit from explicit learning (Maxwell et al., 2017), therefore, it is possible that a more explicit strategy provided an asset in the early years of education, helping these young musicians to improve quickly, even if the strategy is not advantageous long-term, especially after the basics of the skill are acquired. Additionally, in late starters, peer pressure also might play a role in playing complex materials early: observing the accomplishments of other students of the same age might prompt both the teacher and the student to try to “catch up.”

What is clear, however, is that there are neurological consequences of the late start: the networks underpinning the complex movements needed for playing are developing more optimally if the individual starts playing before the age of seven (Altenmüller et al., 2014), therefore, the late start has been identified as a risk factor for developing MFD (Schmidt et al., 2013; Altenmüller et al., 2014).

In summary, playing challenging materials without fully mastering the required motor skills is a risk factor in developing MFD. The reasons for choosing such a repertoire might be manifold: late start, which can be associated with different learning strategies and possibly peer pressure, or simply, the aptitude of these students. Nevertheless, both a late start and the mismatch between the established motor skills and the requirements of the repertoire seem to put musicians at risk of developing MFD.

### Lack of Encouragement and Authoritative Teaching Style

Apart from how and what is being learned in the educational context, the atmosphere and the teaching style also seem very influential. Items relating to the received encouragement, and positive feedback were found to be significantly different between the groups with musicians with dystonia reporting a less favourable and less supportive environment. Moreover, the SIRS’s subscale, “anxiety around the tutor” approached statistical significance, with the dystonic participants reporting more anxiety around their teachers, which also points toward an adverse experience with the individual tuition. The relationship between motor learning and feedback has been examined in sports settings: Avila et al. (2012) found that encouraging and positive feedback enhances the learning and the performance of the motor task. It also seems logical that if the motor skill is repeated with a negative connotation and emotional content, it might influence the future recall of the skill (Juhan, 2003) as well as the learning process. The link between negative emotions and the onset of the disorder in musicians with already existing anxiety and perfectionism has been suggested; as Altenmüller and Jabusch (2009): “It is possible that emotionally induced motor memory consolidation may facilitate the onset of dystonia in the subgroup of patients with these psychological conditions” (p. 151).

This unfavourable environment was also underpinned by a prescriptive and authoritative teaching style and high demands. Strict constraints on the performance are an inevitable part of playing classical music: many aspects of the performance are subscribed to the player, especially in group settings, such as orchestral or chamber music work, leaving little room for spontaneous changes. These musical constraints make classical musicians more vulnerable to developing MFD than jazz or pop musicians who have more freedom to modify the played material (Altenmüller and Jabusch, 2009).

As inevitable as some restrictions are, there are aspects where the musician should be able to make their own decisions, especially in an educational setting, in order to explore their individual interpretation of the pieces and their technical capabilities (Jørgensen, 2000). The autonomy of the student is an important contributor to optimal learning (Wulf and Lewthwaite, 2016; Katz and Westera, 2019): it has been associated with self-efficacy, motivation, attention, and positive cognitive processes during the task (Lemos et al., 2017).

If the general restrictions of classical music are furthered by the teacher’s expectations, and the students must play in a prescribed way, the required precision also increases, and with it, the risk for developing MFD. The more highly specialized a skill’s neural representations are, the less ability they have to adapt to different task requirements (Sadnicka et al., 2018). Moreover, demanding one specific way of performance and interpretation makes the difference between success and failure even smaller. The neurological consequences of placing demands on students that they are not able to meet have already been discussed, but it also has psychological consequences. Trying to meet unattainable demands can provoke anxiety, which has already been linked to the onset of MFD (Enders et al., 2011), perfectionism, a trait which has been repeatedly reported as a typical characteristic of musicians with MFD (Jabusch and Altenmüller, 2004; Jabusch et al., 2004), and rumination, especially following negative feedback (Nepon et al., 2011).

### Mistake Rumination

There are established links between socially prescribed perfectionism, anxiety, and rumination (Flett et al., 2002), and the importance of the social and interpersonal aspect in developing distressing, intrusive thoughts have been thoroughly discussed in the literature (Nepon et al., 2011). To put this into the context of music performance, we inquired specifically about performance-related mistake rumination and found that musicians with dystonia ruminate over performance mistakes significantly more than their healthy counterparts. It seems feasible that apart from the individual and innate trait of perfectionism which makes the individual more sensitive to negatively perceived feedback (Hewitt et al., 2006), the social environment also plays a role, given that the ruminative thoughts often appear in response to an external trigger. How these distressing events are processed, and the content and amount of rumination are dependent on the levels of social anxiety, but also, on the received feedback following the event (Morgan and Banerjee, 2008). In other words, the already mentioned unfavourable educational context might have contributed to the mistake rumination which developed in the musicians who later were affected by MFD.

### Trauma and Change

We were not able to show any significant differences between the samples, in spite of the fact that preceding traumatic events or significant change have been repeatedly mentioned in the literature (Tubiana, 2003; Schmidt et al., 2013), and also in our preceding interview study (Détári et al., 2022). One possible reason for this is the chosen tool: the Trauma History Screen (THS) is quite general, listing various traumatic events, from which some are not specifically linked to interpersonal relationships or music performance, such as accidents and natural disasters. Also, measuring the frequency of the suffered traumas might not provide sufficient data on the impact they had on the individual. It is clear from the literature about Post Traumatic Stress Disorders (PTSDs), that personality, gender, genetic predisposition, and the available support system can all alter how much the individual is affected (Broekman et al., 2007; Elwood et al., 2009). A recent paper (Alpheis et al., 2021) compared the childhood experiences of musicians with and without MFD using the Adverse Childhood Experiences Scale (ACE-S) and found that MFD sufferers experienced more emotional neglect during their childhood; this research tool specifically focuses on the family environment and does not include any other potential sources of trauma. Therefore, it seems that the early development and experiences in terms of trauma can be risk factors, however, other types of traumas are not significant predictors in the development of the disorder. We also added a self-constructed scale, listing significant events which were found to be influential by the interview study. These included change in marital status, jobs, or teachers, becoming a parent, death of a close relative and similar items. We, again, were not able to show any significant differences in the groups. One reasonable explanation for this is that musicians with dystonia were asked to report about the year prior to the onset, while the healthy sample about the previous year. Since we administered the survey while the COVID 19 pandemic was still significantly influencing the lives of performing musicians, many healthy participants experienced more life-altering changes than they would have under normal circumstances.

In conclusion, while there is a growing amount of literature on the psychological and behavioural triggering factors in MFD, these have not yet been explored in a social and playing specific context. While this study has not been able to show very strong and conclusive evidence, it still seems important to examine the developmental trajectories of these risk factors. This approach can not only enhance music educational practices in a broader sense but can specifically inform preventative strategies. It also has the added benefit of changing the narrative around the sufferers of the condition and shifting it toward a more compassionate and holistic understanding of the personal experience of the musicians.

### Limitations

The most obvious limitation of this study is that it uses to some degree self-constructed, previously not tested scales. We had to proceed with this method because of the lack of scales measuring the constructs highlighted in the interviews. Therefore, the development and validation of scales measuring the quality of the received music education are suggested to create a more reliable and valid understanding of the phenomenon.

Only 63.8% of the musicians with MFD were diagnosed by a medical professional, therefore, we cannot state with complete confidence that all the participants who reported having MFD are indeed experiencing symptoms of the disorder and not some other form of performance-related problem. However, as more and more information is available describing the condition’s unique and peculiar symptoms, we decided to include all participants in the analysis who claim to have MFD.

Also, musicians with MFD were asked to recall personal memories, feelings, and psychological states prior to the onset. This was necessary because we were interested in contributing factors, but obviously, it might not be fully reliable and carries possible personal bias. Moreover, there might be some other underlying psychological constructs that are contributing to these differences; systematic and holistic research including all these factors could offer further clarification. Nevertheless, this exploratory study highlights the importance of researching not only currently present traits in sufferers but also the developmental trajectory of these.

## Conclusion

In conclusion, mistake rumination and playing challenging materials prematurely possibly contribute to the onset of the disorder. Moreover, we found significant differences between the education of musicians with MFD and healthy musicians: it seems that those who developed the disorder learned less about healthy technique, were encouraged less, more demands were placed on them, and the received tuition followed a more authoritative style.

The musicians’ lives who are struggling with the condition have to be viewed as complex, continuously evolving systems that are influenced by the environment. This can help us to clarify the aetiology of the condition and can inform preventative strategies.

This is only an early exploration, the first step toward these goals. We need more refined methodologies, to assess various contributing factors and more research to explore how these might impact the individual musicians. Also, as our second interview study (Détári and Egermann, in press) shows, these characteristics and experiences greatly influence rehabilitation as well, so a clearer understanding seems essential in order to enhance the treatment strategies and provide better care for this vulnerable population.

## Data Availability Statement

The raw data supporting the conclusions of this article will be made available by the authors, without undue reservation.

## Ethics Statement

The studies involving human participants were reviewed and approved by Arts and Humanities Ethics Committee, University of York, United Kingdom. The patients/participants provided their written informed consent to participate in this study.

## Author Contributions

AD recruited the participants, collected the data, and conducted the initial analysis and wrote the first draft of the manuscript which was then amended following HE’s feedback and suggestions. HE was overseeing the final analysis. Both authors contributed to the study design and approved the submitted version.

## Conflict of Interest

The authors declare that the research was conducted in the absence of any commercial or financial relationships that could be construed as a potential conflict of interest.

## Publisher’s Note

All claims expressed in this article are solely those of the authors and do not necessarily represent those of their affiliated organizations, or those of the publisher, the editors and the reviewers. Any product that may be evaluated in this article, or claim that may be made by its manufacturer, is not guaranteed or endorsed by the publisher.
