# Supplementary material for: Towards a Holistic Understanding of Musician’s Focal Dystonia: Educational Factors and Mistake Rumination Contribute to the Risk of Developing the Disorder
Source: Front Psychol. 2022 May 9;13:882966. doi: 10.3389/fpsyg.2022.882966 (PMC9125209; doi:10.3389/fpsyg.2022.882966)
Supplement: Supplementary file 1 [file Data_Sheet_1.pdf]

Appendix A.

Self-constructed questionnaire – original grouping of the items

I.	Technique focused teaching
	1. In the lessons, most of the time was spent on small segments of the technical aspects of the playing. (Technical focus)
	2. My teacher attempted to change my technique. (Changed technique)
	3. My teacher allowed me to play with my own individual technique without attempting to change it. (Own technique) Reversed
	4. My teacher always made me focus on musical expression. (Expression) Reversed
II.	Body mechanics
	1. My teacher never paid attention to my posture or corrected when necessary. (No posture)
	2. My teacher did not care if I played with a healthy technique as long as I got the notes right. (No health support)
	3. My teacher was concerned if I had any pain or discomfort while playing. (Concern over discomfort) Reversed
	4. My teacher frequently spent time explaining how to use my body when playing to avoid injuries. (Avoid injuries) Reversed
III.	Socially prescribed perfectionism/ High demands
	1. I always felt like that my teacher asked for more than I was capable of. (Too much asked)
	2. During my lessons, I felt I had to get things right on the first try. (First try)
	3. My teacher was always encouraging when I made mistakes. (Encouragement) Reversed
	4. My teacher always gave me time to figure out a new piece, exercise, or technique. (Give time) Reversed
IV.	Authoritative teaching style
	1. I was unsure what was expected of me. (Unsure expectations)

	2. All aspects of the performance (e.g., dynamics, tempo, articulation) were prescribed by my teacher. (Prescription)
	3. My teacher's instructions were always clear. (Clear instructions) Reversed
	4. My teacher let me develop my own approach to each piece I played. (Own approach) Reversed
V.	Technical problems
	1. I had ongoing difficulty with certain aspects of my instrumental technique. (Technical difficulty)
	2. No matter how much I practised; I was still unsatisfied with some technical aspects of my playing. (Unsatisfied)
	3. My teacher always found a way to help me solve my technical issues. (Solving issues) Reversed
	4. With enough practice, I was always able to solve any technical challenges. (Self-efficacy) Reversed
VI.	Early success
	1. I often played music which was ahead of my class. (Early success)
	2. When I started the instrument, I improved quicker than my classmates. (Quick improvement)
	3. Playing was relatively easy for me at the start. (Easy start)
	4. I entered competitions, got selected for special positions, won auditions very early during my years of study. (Ahead of class)

Appendix B.

Items in the self-constructed scale's factors

Factor 1.	Health and encouragement
	1. My teacher never paid attention to my posture or corrected when necessary. (No posture)

	2. My teacher always made me focus on musical expression. (Expression) Reversed
	3. My teacher frequently spent time explaining how to use my body when playing to avoid injuries. (Avoid injuries) Reversed
	4. My teacher did not care if I played with a healthy technique as long as I got the notes right. (No health support)
	5. My teacher always gave me time to figure out a new piece, exercise, or technique. (Give time) Reversed
	6. My teacher’s instructions were always clear. (Clear instructions) Reversed
	7. My teacher was always encouraging when I made mistakes. (Encouragement) Reversed
	8. My teacher was concerned if I had any pain or discomfort while playing. (Concern over discomfort) Reversed
	9. My teacher always found a way to help me solve my technical issues. (Solve issues) Reversed
Factor 2.	Instrumental technique
	1. In the lessons, most of the time was spent on small segments of the technical aspects of the playing. (Technical focus)
	2. My teacher allowed me to play with my own individual technique without attempting to change it. (Own technique) Reversed
	3. I had ongoing difficulty with certain aspects of my instrumental technique. (Technical difficulty)
	4. My teacher attempted to change my established technique. (Changed technique)
	5. My teacher let me develop my own approach to each piece I played. (Own approach) Reversed
Factor 3.	Demands and authoritative teaching
	1. I always felt like that my teacher asked for more than I was capable of. (Too much asked)
	2. During my lessons, I felt I had to get things right on the first try. (First try)

	3. All aspects of the performance (e.g., dynamics, tempo, articulation) were prescribed by my teacher. (Prescription)
